# Promoting the empowerment and emancipation of community-dwelling older adults with chronic multimorbidity through a home visiting programme: a hermeneutical study

**DOI:** 10.1186/s12912-024-02117-2

**Published:** 2024-06-28

**Authors:** Iria Dobarrio-Sanz, Anabel Chica-Pérez, Olga María López-Entrambasaguas, José Manuel Martínez-Linares, José Granero-Molina, José Manuel Hernández-Padilla

**Affiliations:** 1 Department of Nursing, Physiotherapy and Medicine, University of Almeria, Almeria, 04120 Spain; 2 Emera Nursing and Residential Home for Older Adults, Almería, 04007 Spain; 3 Nursing Department, Faculty of Health Sciences, University of Jaén, Jaén, Spain; 4 Nursing Department, Faculty of Health Sciences, University of Granada, Granada, Spain; 5 Facultad de Ciencias de la Salud. Universidad Autónoma de Chile, Santiago de Chile, Chile

**Keywords:** Chronic illness, Autonomy, Experiences, Home visits, Multimorbidity, Older adult, Self-care

## Abstract

**Introduction:**

Nurse-led preventive home visiting programmes can improve health-related outcomes in community-dwelling older adults, but they have not proven to be cost-effective. Home visiting programmes led by nursing students could be a viable alternative. However, we do not know how community-dwelling older adults with chronic multimorbidity experience home visiting programmes in which nursing students carry out health promotion activities. The aim of the study is to understand how community-dwelling older adults with chronic multimorbidity experience a home visiting programme led by nursing students.

**Methods:**

A qualitative study based on Gadamer’s hermeneutics. Thirty-one community-dwelling older adults with chronic multimorbidity were interviewed in-depth. Fleming’s method for conducting hermeneutic, Gadamerian-based studies was followed and ATLAS.ti software was used for data analysis.

**Results:**

Two main themes were generated: (1) ‘The empowering experience of a personalised health-promoting intervention’, and (2) ‘The emancipatory effect of going beyond standardised self-care education’.

**Conclusions:**

The home visiting programme contributed to the community-dwelling older adults feeling more empowered to engage in health-promoting self-care behaviours. It also improved the older adults’ sense of autonomy and self-efficacy, while reducing their loneliness and addressing some perceived shortcomings of the healthcare system.

**Clinical relevance:**

Older adults participating in a home visiting programme led by nursing students feel empowered to implement self-care behaviours, which has a positive impact on their perceived health status. Nurse leaders and nursing regulatory bodies could collaborate with nursing faculties to integrate preventive home visiting programmes led by nursing students into the services offered to community-dwelling older adults with chronic multimorbidity.

**Supplementary Information:**

The online version contains supplementary material available at 10.1186/s12912-024-02117-2.

## Introduction

Chronic multimorbidity is the most frequent health condition among older adults (people aged ≥ 65 years) [1]. The global prevalence of multimorbidity in older adults ranges from 55 to 98% [1]. In Spain, almost 70% of older adults suffer from chronic multimorbidity [2], which is defined as the coexistence of 2 or more conditions of long duration and slow progression [3]. Chronic multimorbidity is associated with poorer health outcomes, higher mortality rates and increased healthcare expenditure [4]. In addition, older adults with chronic multimorbidity have difficulties implementing self-care behaviours, incorporating healthy lifestyle habits and adhering to treatment [5]. Nurses and policymakers should develop strategies that promote autonomy and self-care in this population [6].

## Background

According to the ‘Middle-Range Theory of Self-Care of Chronic Illness’, self-care is a complex process that requires individuals to implement behaviours to maintain well-being (self-care maintenance), to identify signs and symptoms of their chronic conditions (self-care monitoring) and to respond to these signs and symptoms (self-care management) [7]. In order to optimise self-care, people with chronic conditions need to have sufficient knowledge, skills, motivation, social support and access to healthcare [8, 9]. Therefore, nurses play a key role in the implementation of health promotion interventions [10–13] that aim to help people with chronic multimorbidity assume greater responsibility for their own health care, which can in turn enhance their independence and autonomy [8, 14–16].

Evidence suggests that preventive home visits promote biopsychosocial health and self-care behaviours in older adults with chronic multimorbidity [17]. A preventive home visit is a service in which trained healthcare professionals visit individuals in their own home with the aim of increasing their autonomy [18]. In this context, nurses can engage in progressive and individualised health promotion, while increasing the patient’s understanding of their conditions and therapeutic regimen [18]. However, results on the cost-effectiveness of preventive home visiting programmes are inconsistent and this makes it difficult to integrate them into the services offered by healthcare systems [19]. Preventive home visiting programmes with trained volunteers have been tested as an alternative to home visiting programmes run by healthcare professionals, but these have only proven to have a small short-term effect on some self-care behaviours in older people with chronic conditions. Therefore, other interventions should be tested [20]. Research shows that nursing students are capable of effectively implementing individualized health promoting interventions [21–24] that could have positive effects on patients [25]. In fact, other interventions in which nursing students promoted health among vulnerable groups have shown positive effects on quality of life [26], accessibility to the healthcare system [27] and health knowledge [28, 29]. In this regard, nursing students could be a health asset for community-dwelling older adults with chronic multimorbidity [30]. Teaching interventions based on practical experiences have been associated with improved health and satisfaction amongst older adults [25], as well as changes in students’ perceptions and attitudes towards them [23, 31]. Although these findings are encouraging, we do not know how community-dwelling older adults with chronic multimorbidity experience home visiting programmes in which nursing students carry out health promotion activities.

## The study

### Aim

The aim of this study was to understand how community-dwelling older adults with chronic multimorbidity experience a home visiting programme led by nursing students.

### Design

A qualitative study was conducted based on Gadamer’s hermeneutic philosophy. This approach is often used to reach a deep understanding of a phenomenon [32]. According to Gadamer’s hermeneutic philosophy, incorporating one’s pre-understanding into a dialogue with others allows for both perspectives to be merged through a fusion of horizons that can lead to understanding the phenomenon [33]. For methodological consistency with Gadamer’s hermeneutics, this study was conducted following the method developed by Fleming et al. [32]. Firstly, the researchers reflected on the research question’s relevance and agreed that understanding the community-dwelling older adults’ experiences of a home visiting programme led by nursing students is a lifeworld phenomenon that could provide valuable insights for the discipline. Secondly, the researchers’ pre-understanding of the study phenomenon was identified. The researchers’ preconceptions were drawn from their clinical, teaching and research experience with community-dwelling older adults with chronic multimorbidity. In developing the research report, the recommendations of the Consolidated Criteria for Reporting Qualitative Research (COREQ) guide were followed.

### Participants and context

The study was conducted in 10 primary care centres in a province in southern Spain. Inclusion criteria for participation in the study were: (1) to be 65 years of age or older; (2) to have 2 or more chronic conditions; (3) to not be receiving home support through the public or private healthcare system; (4) to have completed a 12-week preventive home visiting programme implemented by nursing students; (5) to have given consent to be contacted for participating in future qualitative studies about their experience of the home visiting programme. Participants were recruited through purposive sampling. The eighty-four older adults who had explicitly given consent to be contacted were invited to participate by the study’s lead researcher (who had not been in contact with them during the home visiting programme). Sixteen older adults declined the offer due to ill health (*n* = 10) or lack of time (*n* = 6).

An in-depth description of the home visiting programme has been published elsewhere [23]. In summary, the home visiting programme lasted 12 weeks. The home visits took place once a week and lasted between 30 and 45 min. The aim of the home visits was to promote health in older adults with chronic multimorbidity. The participants received individualised information tailored to their health situation, needs and preferences. The participants received information on: (1) self-care of the chronic conditions and management of the prescribed treatment, (2) healthy habits (i.e. diet and physical exercise), (3) use of healthcare resources, and (4) autonomy in decision-making and advance directives. Three home visits were carried out for each topic. The first home visit was organised to present the information on the topic, the second to elaborate on it, and the third to reinforce it and evaluate the patient’s progress. The students who carried out the home visits were enrolled in the third year of the Bachelor’s Degree in Nursing at the University of Almería (Spain) and received 40 h of specific training within the Elderly Care module to prepare them for conducting the home visits.

### Data collection

According to the third stage of Fleming et al.‘s method [32], we continued to deepen into the understanding of the phenomenon through a dialogue with the participants’ accounts. Data collection was carried out between January and March 2023 through in-depth interviews that lasted between 40 and 60 min, with an average duration of approximately 48 min. The interviews were conducted by two researchers with a specialist diploma in qualitative research methods as well as experience in conducting in-depth interviews. The participants were contacted via a phone call, during which the name and objective of the study were presented. The participants who expressed willingness to participate were contacted again to arrange an appointment for a face-to-face interview in their homes. The interview script was specifically developed for this study and designed in such a way that would allow participants to give in-depth responses (Supplementary file 1). The script was rehearsed before the interviews took place, and the two interviewers ensured that the interview was conducted to feel like a conversation. All interviews began with the following question: “Please tell me about your experience with the home visiting programme.” The interviews were audio-recorded with the prior consent of the participants. All transcripts were returned to the participants for confirmation and no corrections were made. When 31 interviews had been conducted, the researchers considered that data saturation had been reached and data collection was stopped because the information was redundant or did not add anything new.

### Data analysis

All field notes and individual interviews were transcribed into a text document for further analysis using ATLAS.ti software. For data analysis, the method developed by Fleming et al. was followed [32]. The first stage of the data analysis process focused on gaining an understanding of the phenomenon through a spoken dialogue between the researchers and the participants. The second stage of the data analysis process aimed at reaching a deep understanding of the phenomenon by putting the researchers’ pre-understanding into a dialogue with the text (i.e. the participants’ transcribed accounts). In the second stage, three researchers completed an open and comprehensive reading of the transcripts in order to develop an overall impression of the participants’ experiences. Then, the three researchers individually exposed and identified the meaning of every sentence in the transcript by assigning them codes. The codes that shared patterns of meaning were grouped into ‘units of meaning’. In applying the hermeneutical circle, the ‘units of meaning’ were related to the text as a whole to create subthemes that were subsequently related to the text as a whole to develop themes. As the last step of the data analysis process, the passages that best illustrated the shared understanding between the researchers and the participants (i.e. fusion of horizons) were selected to be included in the manuscript.

### Ethical considerations

This study was conducted in accordance with the ethical principles of the Declaration of Helsinki. All participants were informed of the voluntary nature of their participation and the commitment to confidentiality and anonymity. Signed informed consent was obtained from all participants prior to the start of data collection, which explained the purpose of the study, the nature of their participation and their right to withdraw at any time. Approval was obtained from the Ethics Committee of the Department of Nursing, Physiotherapy and Medicine of the University of Almeria in December 2020 (EFM89/2020).

### Rigour

In line with the final stage of Fleming et al.‘s method [32], methodological rigour was ensured at each stage of the study following Guba & Lincoln’s quality criteria [34]. Credibility was achieved through prolonged engagement (the interviewers were volunteers for a local non-governmental organisation and were familiar with the participants’ situation), collection of referential adequacy material (the interviewers uploaded their interviews, their memos and their field notes to the study’s cloud) and peer debriefing (the research team met with two independent experts who reviewed and provided feedback about the study’s methods and results). To ensure confirmability, the participants confirmed the transcripts and then verified the results. To ensure reliability, data interpretation was supported by researcher triangulation, and the analysis process was evaluated by two independent reviewers. A detailed description of the participants, the context and the method were provided to maximize transferability.

## Findings

The demographic characteristics of the 31 older adults who participated in the study are summarised in Table 1. The mean age of the participants was 75.4 years, and the majority (58%) were female. Participants had a mean of 4.45 chronic comorbidities.

**Table 1:** Main sociodemographic characteristics of the participants

Participant	Age	Gender	Education level completed	Former occupation	Marital status	Number of chronic conditions
P1	82	Male	Secondary	Fisherman	Married	4
P2	78	Female	None	Homemaker	Married	5
P3	70	Female	Primary	Butcher	Married	5
P4	68	Female	None	Cleaner	Widowed	4
P5	65	Female	None	Homemaker	Married	3
P6	82	Male	Primary	Construction worker	Married	6
P7	87	Female	None	Cleaner	Widowed	7
P8	71	Female	None	Homemaker	Married	5
P9	75	Male	None	Construction worker	Married	3
P10	70	Female	Secondary	Homemaker	Married	4
P11	69	Male	Primary	Factory worker	Married	4
P12	82	Male	None	Fisherman	Single	5
P13	69	Male	None	Farmer	Married	4
P14	75	Female	None	Homemaker	Widowed	8
P15	70	Female	Primary	Supermarket assistant	Married	3
P16	88	Male	None	Construction worker	Married	4
P17	85	Female	Primary	Homemaker	Widowed	5
P18	68	Male	None	Waiter	Married	7
P19	72	Female	Primary	Farmer	Married	5
P20	69	Male	Secondary	Telephone technician	Married	4
P21	70	Female	Primary	Childminder	Widowed	4
P22	73	Male	Primary	Farmer	Married	5
P13	78	Male	Primary	Customs worker	Married	3
P24	68	Female	Secondary	Accountant	Single	2
P25	85	Female	None	Business owner	Married	6
P26	68	Female	University	Lawyer	Widowed	3
P27	83	Female	Primary	Tailor assistant	Married	4
P28	75	Male	University	Engineer	Married	3
P29	67	Male	Secondary	Business owner	Married	7
P30	84	Female	None	Housewife	Married	4
P31	91	Female	None	Cleaner	Married	2

Six sub-themes grouped into two main themes were generated during the data analysis, which helped to understand community-dwelling older adults’ experiences of a preventive home visiting programme implemented by nursing students (Table 2).

**Table 2:** Themes, subthemes and codes developed during analysis

Theme	Subtheme	Units of meaning
**The empowering experience of a personalised health-promoting intervention.**	When becoming aware of one’s own situation promotes self-care.	Recognising risk behaviours, health situation awareness, personal vulnerability, feeling empowered, setting new goals.
	Connecting to learning through meaningful self-care advice.	Physical activity and nutrition, holistic health, symptom management, how treatment works, symptom monitoring, support resources, advance directives, self-care technical procedures, applicable advice, practical education.
	“*It’s not too late*”: Incorporating health-promoting self-care behaviours into one’s routine.	Healthier diet, exercising, quitting smoking, reducing alcohol consumption, improving hydration, autonomous medication management, using new-technologies, detecting life-threating complications.
**The emancipatory effect of going beyond standardised self-care education.**	Filling a void left by the public healthcare system.	Professional-centred healthcare system, insufficient consultations, opportunity to talk, companionship, practical health education, answering queries.
	When being heard feels good.	Being heard, feeling the focal point of attention, increased sense of purpose, contributing to nurses’ education, feeling good.
	Reducing vulnerability through self-care education.	More energetic, feeling stronger, higher self-efficacy, better supported, feeling less anxious, increased social participation, improved overall health, increased sense of independence, feeling more autonomous.

### The empowering experience of a personalised health-promoting intervention

The home visiting programme led by nursing students empowered the community-dwelling older adults to implement health-promoting self-care behaviours. Most of the participants’ narratives revolved around the fact that receiving individualised health-promoting education helped them to become fully aware of their own health situation. The individualised nature of the home visiting programme meant that the participants immersed themselves in a learning experience that contributed towards them feeling more independent and autonomous. Most of the participants explicitly mentioned that the home visiting programme led by nursing students showed them that it was not too late to make changes to their lifestyles. Although some participants were more sceptical about the positive impact of their lifestyle changes, all of them stated that they have incorporated health-promoting self-care behaviours into their daily routines.

#### When becoming aware of one’s own situation promotes self-care

Participating in the home visiting programme helped the older adults to become fully aware that their health behaviours were inappropriate and that their lifestyle habits were having a negative impact on their health. The visiting programme was the first step in changing their health behaviours.

*“Participating in this [home visiting programme] has helped me; mainly to realise the mistakes I made with my condition and that what I was doing was very dangerous for my health.” (P28)*.

The visiting programme helped the older adults to have a better understanding of their health condition and treatment. The insights they gained during the home visits brought the reality of their current health situation to light. Many of the participants also reported that they became more aware of life in general and the importance of self-care for a good quality of life.

*“I have learnt to be more aware of my body, my conditions, my symptoms, as well as to get to know myself better, to reflect, to think about my life, about what I want to do.” (P3)*.

*“This has really helped me to control my conditions, because everything I have learnt makes me more aware of them.” (P9)*.

Furthermore, the number and duration of the visits in the programme allowed the nursing students to address topics in an individualised and tailored manner. As a result of these interventions, the older adults were able to come to terms with the fact that they were ageing and link the changes they were experiencing in their bodies to this process. Awareness of their vulnerability made them take an interest in maintaining good health and acquiring effective self-care behaviours.

*“I had never had anyone explain everything the way the student did, and it was very good, but it also showed me that I am no longer a 30 something year-old girl. I am 85 and I am not as strong as I used to be. My joints ache, a simple cold can make me feel very sick for days on end… I really need to be careful”. (P25)*

Empowering patients is the process of helping them to become aware of their decision-making capacity, thus freeing them from coercion or impositions. It also involves supporting them in making the best possible decisions in terms of self-care, which can reduce their dependence on healthcare providers. The participants felt empowered and competent to make appropriate decisions about their health and their lives. The visiting programme motivated them to learn self-care behaviours to improve or maintain their health.

*“Well, I feel that I can manage my condition better, I feel like I can have more control over my life in general when I do things that are good for my conditions.” (P5)*.

The student nurses were key figures in empowering the older adults. The home visiting programme encouraged the student nurses to get to know the older adults well enough to help them set achievable and progressive goals for change. The participants felt that the home visiting programme provided them with the tools they needed to change their health behaviours. Many of the older adults explained how they had already incorporated new health behaviours into their daily lives to fulfil their health objectives, even if they were doubtful about whether they would have a positive effect on their health.

*“I have decided that I want to get stronger. I was in good shape when I was young, but I stopped working and I got fatter and then everything started to hurt more […] I know the exercises that will help me, and I am going to get stronger again.” (P11)*.

*“I am not sure this is going to help at my age, but I am still doing what the student recommended me […] I am old and sick…” (P16)*.

#### Connecting to learning through meaningful self-care advice

The visiting programme enabled the older adults to acquire the knowledge and skills necessary to improve their self-care. The participants explained how exposure to specific and individualised learning in the different sessions empowered them to eat healthy meals and exercise, all within their limitations. A few participants felt that the lifestyle changes recommended by the nursing students were hard to implement. Nonetheless, receiving health-promoting education allowed most of the older adults to connect with the learning process because they saw that what they had been taught was directly applicable to their day-to-day life. The participants became aware of the importance of holistic health; they understood that every part of their body was connected and that a bad habit could have negative consequences on more than one of their conditions.

*“I have learnt all sorts of things: how to do Kegel exercises, how to find recipes for cooking the food I like in a healthier way, and I am enjoying that. It keeps me busy.” (P24)*.

In relation to the learning process, the participants felt that they had the necessary tools and knowledge to maintain, monitor and manage self-care. Three older adults admitted to not having interest in furthering their understanding of their pharmacological treatment, so they only focused on learning to manage their own monthly pill box autonomously. However, most participants’ learning went beyond organising their pill box or identifying which medication was required for a particular condition. In fact, many of the older adults mentioned that the visits focusing on medication management helped them to get to know how to handle adverse situations related to their treatment, such as what to do if they forgot a dose. The participants also felt able to manage exacerbations of their chronic conditions, and competent enough to discern whether they needed to visit a healthcare centre.

*“At my age it is normal to have some problems here and there [participant smiles], but I have learnt what to look for when I feel unwell and to decide whether it is serious.” (P31)*.

The home visiting programme helped the older adults to navigate the healthcare system as they became familiar with the wide variety of support resources available to them that they had not been aware of before. Many of the participants felt satisfied with knowing about a range of community programmes where they could spend their time.

*“It has helped me to find out about the support available to me, aside from the healthcare centre, the hospital and nursing homes. There are loads of things I can do that nobody had told me before.” (P28)*.

The home visiting programme made the older adults aware of advance directives. Most of the participants found this subject interesting, and some of them even started the process after the visiting programme. However, death is still considered a taboo subject for many of the older adults. In fact, some of them felt uncomfortable during the visits related to advance directives and even thought it was useless to set them up.

*“What interested me the most was the advance directives. I didn’t know they existed, and I think they are very important for people who are nearing the end of their lives. I have started the process to sort mine out.” (P31)*.

*“I am afraid of having a will, I didn’t know about it. I don’t like it. If you go into a coma and you want to die, you write it down and they don’t do anything… you will be dead…” (P18)*.

The nursing students organised the contents of the sessions to promote practical health education. As a result, the older adults felt that all the advice and recommendations could be progressively applied to their daily lives, which boosted their motivation to change their self-care behaviours and improve their health.

*“It was easy to follow the advice she gave me (the student nurse) because she never asked me to do big things. We focused on one or two things every week.” (P17)*.

#### “It’s not too late”: incorporating health-promoting self-care behaviours into one’s routine

According to most participants, the home visiting programme helped them to realise that it is never too late to learn and to incorporate health-promoting self-care behaviours into their daily routine. Following the home visiting programme, even the older adults who thought that it was *“[…] a bit late to start being super healthy… (P16)”* did indeed make lifestyle changes. For some it was related to their diet, while others mentioned feeling motivated to exercise or even reduce their tobacco and alcohol consumption. The nursing students have encouraged the implementation of self-care behaviours through very simple interventions adapted to the older adults’ needs, such as having a jug of water on the table to stay hydrated.

*“It’s not too late. That’s the most important lesson I’ve learnt. She has motivated me to exercise, which I do every day, and it’s like I feel better in general.” (P14)*.

*“If the student hadn’t come, I would still smoke a packet or a packet and a bit [a day] and now I smoke half a packet.” (P4)*.

After the home visiting programme, the older adults improved their medication management as they became more familiar with their regular treatment. They alluded to better medication adherence due to the various strategies and resources provided by the nursing students during the home visiting programme, such as the use of pill boxes and planning alarms on their smartphones.

*“I didn’t really know what all the different medicines were for, and I used to take them at any time of the day, but now I know how to organise my medicines better with a pill box and alarms on my mobile phone.” (P29)*.

The home visiting programme facilitated the integration of new technologies as a tool to support self-care; the older adults used technology to search for strategies and recommendations to improve self-care behaviours. The visiting programme was also useful in bridging the digital divide and avoiding the associated social isolation. As a result, the older adults experienced increased self-esteem and social integration.

*“I have downloaded so many apps for my smartphone… I am constantly looking for new recipes, to check my progress, to see how much water I have drunk and all those things because it keeps me entertained as well.” (P28)*.

The home visiting programme also provided some of the older adults with the skill of identifying life-threatening complications. This key self-care behaviour enables them to react quickly to reduce the severity of adverse health situations.

*“I have learnt many things, (…), what I must do when something happens to me like in the case of stroke, smiling [look in the mirror and smile], raising my arm… I didn’t know about these things before, and they helped me to identify it when it happened to me, and I went to hospital straight away”. (P20)*

### The emancipatory effect of going beyond standardised self-care education

The community-dwelling older adults participating in the study compared the student-led home visiting programme with the care provided by nurses and physicians in the public healthcare system. The participants believed that the home visiting programme led by nursing students filled a void left by the public healthcare system; not only did it deliver tailored health-promoting education, but it was also designed in a way that the older adults always felt at the centre of the care provided. They believed that they were contributing to society by helping with the education of future nurses, which increased their sense of purpose. Making self-care advice meaningful for the participants made them more engaged when learning new health-promoting behaviours, which they incorporated into their daily routines. Consequently, the older adults believed their biopsychosocial well-being, independence and autonomy improved after participating in the home visiting programme led by nursing students.

#### Filling a void left by the public healthcare system

All older adults participating in the study spontaneously compared the intervention led by nursing students with the usual care they receive from the public healthcare system. The participants feel that the healthcare system does not meet their needs as it is more focused on the healthcare professionals than on the patients. In fact, after the home visiting programme, many of the older adults were more aware of the shortcomings of the healthcare system and the lack of individualised care provided. Furthermore, they felt that they were able to address their health concerns effectively during the programme, unlike with usual health care where time constraints can be an obstacle.

*“At the healthcare centre they just tell me “you have this, so take this and that, at such and such a time.” Here they explained things to me calmly, in more depth and in such a clear way that it was perfectly understood.” (P27)*.

The home visiting programme enabled the participants to establish a relationship of trust with the nursing students. They felt that they had a safe space to disconnect and discuss issues and concerns that they would not normally broach with anyone else. The older adults said they looked forward to each session because they felt supported. In fact, after the home visiting programme, the participants expressed how much they were going to miss the company of the nursing students.

*“It has been good for me to spend more time with someone, to talk for longer and to share my thoughts and talk about my concerns calmly. I never used to talk about these issues and it’s been great having these sessions every week.” (P4)*.

*“I wish they had left her [the student nurse] longer and that it wasn’t over, because I spend the whole week waiting for her to come because I have felt so relieved lately, and I will miss her company.” (P15)*.

The older adults appreciated the effort made by the nursing students to put them at ease and promote effective self-care behaviours. They felt grateful for the enthusiasm and eagerness with which they carried out each of the sessions.

*“I noticed on each home visit that he [the student nurse] put a lot of effort into it and it was really splendid.” (P27)*.

Furthermore, all the older adults mentioned that they were very pleased with the student nurses because they adapted their explanations to the socio-cultural level of the participants and answered their questions as many times as necessary. The home visiting programme offered the older adults the opportunity to address almost all their concerns and queries, as long as they fell within the nursing students’ competency remit.

*“I liked it a lot because I asked a lot of questions, and the student answered me very calmly in a way I understood.” (P27)*.

#### When being heard feels good

The participants felt heard and that their problems were taken seriously throughout the individualised student-led home visiting programme. Most of the older adults highlighted the interest shown by the student nurses and how they felt confident enough to discuss any issue with them. Feeling heard made them feel good.

*“She [the nursing student] was so warm the whole time. She was very patient, she listened to my concerns… I felt very good because I was comfortable with her.” (P30)*.

Due to the content and holistic nature of the sessions, the home visiting programme made the participants feel that they were the focal point of attention. It also increased their sense of purpose as they recognised that they were helping the nursing students to gain experience and further their education. This symbiotic relationship made the participants willing to relive the experience.

*“I would repeat the experience not only for myself but also for the students. I like helping them gain experience.” (P15)*.

The participants were grateful for the experience and aware of their contribution to nursing education; the home visiting programme was an opportunity for nursing students to learn how to care for older adults and join the workforce as competent nursing professionals.

*“And thank you to the professors who had the idea to do this because it gave us the opportunity to show future nurses what problems older people have so they know how to look after us when they finish their studies. (P22)*

#### Reducing vulnerability through self-care education

The home visiting programme helped the participants to feel more energetic, more active and stronger, which made them capable of doing activities that they did not do before. As a result, they felt more useful.

*“I feel stronger and much better and now I do things I didn’t used to do, like help in the kitchen or in the garden.” (P3)*.

The nature of the home visiting programme promoted greater self-efficacy for self-care in most of the participating older adults, who stated how their attitude towards changing inappropriate health behaviours had shifted. The home visiting programme has increased their confidence and self-esteem in making decisions about their health.

*“I feel more confident because I have learnt to do things that I didn’t do before, and above all because it has helped me to acquire skills and adapt activities that I was doing incorrectly.” (P31)*.

The sessions focusing on the available community resources facilitated the older adults’ access to them. After the home visiting programme, many of the participants mentioned that they had gone to different associations in their area and were participating in programmes that increased their level of social interaction. As a result, the older adults perceived higher social support and felt less vulnerable.

*“I didn’t know they offered help in the Red Cross for example. I feel that people care about older people like us and that it is nice because I can now call different places and they will help me if I need it.” (P18)*.

Nonetheless, the home visiting programme implemented by nursing students was not enough to increase the social participation of some older adults, who felt overwhelmed by the idea of having to interact with people they did not know.

*“For me it is a bit too much, to be honest* [enrolling in activities for older adults]. *I like being at home doing my own things […] I’ve always been at home looking after my husband, my children, my house tasks…” (P14)*.

The participants mentioned an improvement in their mental health thanks to the home visiting programme; they felt less anxious and were in a better mood from the very start. Many of them also have a more active lifestyle now. As the home visiting programme led the older adults to join various programmes, their social participation increased as a result.

*“I don’t think about silly things anymore. It has helped me to encourage myself to go for a walk and clear my head. And even if I don’t feel like it, I force myself to talk and socialise with my friends.” (P19)*.

Following their participation in the home visiting programme, the older adults felt that their general health had improved; they talked about having better control of their conditions and improved general wellbeing.

*“It’s incredible. I don’t know why but I feel much better… I have got less reflux, I feel lighter and I feel healthier.” (P22)*.

The home visiting programme helped the older adults to gain independence; they felt able to perform certain basic activities of daily living independently, for which they previously needed help. The home visiting programme made them feel useful, changed their self-perception, and improved their autonomy.

*“At first I thought it [the home visits] wouldn’t be important, but when I stopped to think about it and analyse everything, I see that everything the student explained to me is useful and it helps me to do things on my own. I feel more independent.” (P9)*.

*“I have learnt a lot but the most important thing for me is that I feel she [the nursing student] has helped me to realise that I can make my own decisions. Yes, I am going to listen to my family and the doctors but if I don’t want to do something, I don’t want to… It’s my decision.” (P27)*.

## Discussion

The aim of our study was to understand community-dwelling older adults’ experience of a home visiting programme led by nursing students. The results of the study suggest that the experience of participating in such a programme improves the community-dwelling older adults’ self-confidence and self-care skills, which are acquired through individualised and tailored health education interventions carried out by nursing students. According to other studies, this could result in perceived improvements in the older adults’ own health self-management [35], improved quality of life [18], and increased satisfaction and confidence [25]. The home visiting programme encouraged the older adults to learn more about their overall health and initiate changes in their health behaviours. Furthermore, it had a positive impact on the way they saw themselves, contributing to their biopsychosocial well-being, independence and autonomy. Our results suggest that it also made the older adults aware of the limitations associated with the ageing process. According to the experiences of other older adults participating in a nurse-led health promotion intervention programme, recognising one’s health status promotes self-care behaviours [36]. Similarly, health literacy is a direct and robust predictor of self-care behaviours [37]. Therefore, the acquisition of self-care behaviours could have been achieved through the home visiting programme’s objective to promote health in older adults with chronic multimorbidity. In addition, the home visiting programme provided individualised care tailored to the needs of each older adult, in line with the notion that patient-centred care makes the results of interventions more effective [38]. Our results suggest that the home visiting programme empowered the older adults to make decisions. The older adults’ home is the ideal setting for health promotion because of the meaning it has for the person living in it and as it facilitates the nurse’s contributions [39]. Nonetheless, there were topics addressed during the home visits that elicited conflicting experiences among the participants. Most of the older adults were open to discussing topics related to death and advance directives, experiencing similar feelings to the older adults in a study conducted in Shanghai [40]. However, some of the participants in the study were still reluctant to talk about death, perhaps because awareness of advance directives among older adults is relatively low, partly due to their low educational level [41]. Our results suggest that the nursing students provided highly practical health education through the home visiting programme, which may have made it easier for the older adults to apply what they learned to their daily routine, thus resulting in improved perceived health-related outcomes [42]. The home visiting programme contributed to maintaining the older adults’ motivation to change inappropriate health behaviours. Consistent with the results of other studies, the participants also stated that they had learnt more about their medication and overcome the typical difficulties that older adults with chronic multimorbidity have in adhering to their treatment [5]. The community-dwelling older adults were also provided with physical exercise guidelines, which led to improvements in their biopsychosocial functioning [43]. In the same vein, the participants improved their dietary knowledge, which has been shown to promote adherence to a suitable diet [44]. In addition, our results suggest that the visiting programme could be a way to bridge the digital divide that affects older adults. According to our findings, the older adults’ motivation for using technology is driven by their interest to monitor their health, stay active and access the health system through different mobile applications [45]. Moreover, evidence suggests that the use of technology increases older adults’ self-efficacy [46]. Our results also suggest that the home visiting programme itself provides adequate self-efficacy strategies. According to one study, individuals with higher self-efficacy are more likely to engage in self-care behaviours [47]. In addition, considering the definition of self-care provided by the ‘Middle-Range Theory of Self-Care of Chronic Illness’ [7], the home visiting programme enabled the older adults to detect life-threatening complications.

The home visiting programme not only gave the older adults the opportunity to empower themselves and acquire certain self-care behaviours, but also exposed the shortcomings of the public healthcare system. The organisation of healthcare systems and the high caseload for healthcare professionals often require older adults to be seen in very short time slots [48]. This makes it difficult for the healthcare professional to answer questions and provide recommendations tailored to the needs of the individual patient [49]. The participants alluded to the fact that the home visiting programme is a health resource that goes beyond the range of services offered by the public healthcare system. The participants perceived the care received from student nurses much more positively than the care provided by professionals in healthcare centres. The community-dwelling older adults valued the time the student nurses dedicated to explaining their condition without rushing, as well as their calm and friendly demeanour. Both communication problems and the lack of sufficient time spent with patients have already been highlighted in other research [50]. Sufficient care time is a necessary care requirement for older adults with multimorbidity [51] and is one of the factors most valued by patients [50]. Our results suggest that the home visiting programme allowed for the provision of patient-centred care and the establishment of trusting relationships between the older adults and nursing students. According to available evidence, patient-centred care is essential in the acquisition of self-care behaviours and shared decision-making [52]. Consistent with our results, a trusting relationship can improve older adults’ mood and reduce feelings of loneliness [53]. Furthermore, our findings suggest that the visiting programme made the older adults feel like useful members of society by contributing to nursing education. In fact, another study conducted with nursing students indirectly evidences the importance of older adults in the personal and professional education of future nurses [23]. Finally, our results show that the older adults’ experience of a home visiting programme contributed to improved empowerment and self-care skills, which had a positive impact on their overall health.

## Limitations

This study has some limitations. Firstly, in relation to the selection of the sample, the older adults who were interviewed were not receiving support from the Spanish Dependency Law, so they did not have any other standardised home care. This may have influenced their perceptions of the healthcare system. A limitation in terms of data collection could be that only one interview was conducted at the end of the programme. Carrying out multiple interviews throughout the programme would have provided information on how the older adults’ perceptions might have changed at different stages in the programme. Including participant observation could have provided information on how a particular visit determines the patients’ post-visit behaviours. Lastly, it is worth noting that the home visits were conducted just after COVID-19 restrictions took place, which limited face-to-face care in healthcare centres as it was replaced by telecare. This may have conditioned the older adults’ perceptions of the care provided by the healthcare system.

## Conclusions

The preventive home visiting programme led by nursing students has contributed to the community-dwelling older adults feeling more empowered to engage in health-promoting self-care behaviours. The home visiting programme could not only reduce perceived loneliness in community-dwelling older adults with multimorbidity, but it could also increase their perceived autonomy and self-efficacy to implement health-promoting self-care behaviours. The older adults participating in this study considered that the preventive home visiting programme led by nursing students filled a void in the standard care offered by healthcare systems. Future studies could explore the design of policies that promote the integration of nursing students as a potential health asset for community-dwelling older adults with chronic multimorbidity.

### Electronic supplementary material

Below is the link to the electronic supplementary material.

Supplementary material 1

## Data Availability

The datasets used and/or analysed during the current study are available from the corresponding author on reasonable request.
