# Supplementary material for: Promoting the empowerment and emancipation of community-dwelling older adults with chronic multimorbidity through a home visiting programme: a hermeneutical study
Source: BMC Nurs. 2024 Jun 28;23:444. doi: 10.1186/s12912-024-02117-2 (PMC11212443; doi:10.1186/s12912-024-02117-2)
Supplement: Supplementary file 1 — Supplementary material 1 [file 12912_2024_2117_MOESM1_ESM.docx]

**Supplementary file 1.** Interview script.

| **Phase** | **Topic** | **Content/Questions** |
| --- | --- | --- |
| **Presentation** | Purpose | Explain to the participants that their contributions are highly valuable. |
|  | Objective | To understand how community-dwelling older adults with chronic multimorbidity experience a home visiting programme led by nursing students. |
| **Opening** | Opening question | You have participated in a home visiting programme led by nursing students. Please tell me about your experience with the home visiting programme. |
| **Development** | Specific questions | What do you think of the programme you have participated in?  What did you enjoy the most about this experience?  What was the aspect of the programme you enjoyed the least and how would you improve it?  How do you think the programme has helped you? |
| **Closing** | Contributions | Do you think we have left out any important questions or would you like to make any further comments? |
|  | Acknowledgements | We appreciate your willingness to participate. Our study would not be possible without your input. |
